# Proposed Framework for the Psychological Treatment of Munchausen by Proxy Survivors

**DOI:** 10.1007/s10880-026-10128-1

**Published:** 2026-02-13

**Authors:** Mary J. Sanders, Brenda Bursch

**Affiliations:** 1 Department of Psychiatry, Stanford University, Stanford, CA United States; 2 Departments of Psychiatry and Pediatrics, University of California, Los Angeles, Los Angeles, CA United States

**Keywords:** Munchausen by proxy, Factitious disorder imposed on another, Treatment

## Abstract

Munchausen by Proxy abuse occurs when a caretaker with a psychiatric condition called Factitious Disorder Imposed on Another intentionally falsifies illness and/or disability in another, usually a mother falsifying illness or disability in her child. Optimal recovery from MBP abuse requires a variety of interventions with the survivor, with an emphasis on using accurate information to help the survivor better understand reality and develop a healthier self-narrative. The goals of this paper are to provide information about the psychological aspects of Munchausen by Proxy abuse and to propose a psychological treatment framework tailored to the needs of survivors. Components of the heuristic model, using the acronym STORIES, include Safety and Stabilization, Trauma Processing & Support, Obtaining Information; Rebuilding Trust and Relationships; Integration of Health and Well-Being; Empowerment and Self-Esteem, and Sustained Support and Follow-Up. The model can be used as a guide for mental health providers, child and adult welfare professionals, and legal experts within the field of maltreatment who are developing safety and treatment plans for MBP survivors.

Munchausen by Proxy abuse occurs when a caretaker with a psychiatric condition called Factitious Disorder Imposed on Another intentionally falsifies illness and/or disability in another, usually a mother falsifying illness or disability in her child (American Psychiatric Association [APA], (2022); American Professional Society on the Abuse of Children [APSAC], (2018). A treatment framework targeting abuse perpetrators has been published (Sanders & Bursch, 2020), however, few details are provided related to the treatment of survivors. Psychotherapy to support survivors of Munchausen by Proxy abuse is complex and requires a tailored, multi-faceted approach. The goals of this paper are to provide information about Munchausen by Proxy abuse and to propose a psychological treatment framework tailored to the needs of survivors. Consultation with an expert is also recommended due to the unique aspects of this form of abuse.

## Understanding Abuser Psychopathology

*Factitious Disorder Imposed on Another (FDIA)*, the psychopathology underlying Munchausen by Proxy (MBP) abuse, is compulsively driven by deep-seated emotional needs in the perpetrator for attention, control, and a sense of importance. Perpetrators are consciously aware of their manipulations and deliberately create symptoms or falsify medical histories, but they often fail to appreciate the harm they are causing or the potential lethality of their actions. Due to attachment difficulties, they often lack genuine empathy for their victims despite being able to appear empathic when observed (Adshead & Bluglass, 2005). They typically have comorbid personality disorders or traits, and more than half may also meet diagnostic criteria for the psychiatric diagnosis of *Factitious Disorder Imposed on Self* (APA, 2022; Ayoub, 2010; Bass & Jones, 2011; Bools et al., 1994; Irwin & Bursch, 2021). Diagnostic assessment guidance is available (Bursch et al., 2021a, 2021b).

Despite sometimes being suspected by family members or friends, MBP is most often reported to child or adult welfare professionals by clinicians of the victim. While the presentations vary, MBP can result in permanent harm or death of the victim, either because clinicians use inaccurate information to develop assessment and treatment plans that lead to iatrogenic harm or direct harm due to induced illness or intentional neglect of a genuine medical condition.

## Understanding the Experience of Survivors

Humans have evolved within social groups where cooperation and trust are essential for survival (Bateson, 1988). Storytelling and the sharing of experiences create social connections (Grundel et al., 2007; Gupta & Jha, 2022; Oliver, 2023). A well-told story can trigger oxytocin release, leading to feelings of empathy, care, and connectedness. Thus, an adept storyteller can successfully falsify illness or disability narratives about themselves or others while also being positively regarded.

### Primary Victims

Primary victims are individuals that the abuser falsely claims are more ill or disabled than is objectively true. Complicating the picture, some primary victims of MBP abuse have genuine medical or developmental disorders that are being exacerbated by the abuser (Abraham-Bizot et al., 2023). These disorders or disabilities can be congenital, develop naturally after birth, or be the result of iatrogenic harm (from deprivation or unneeded or incorrect medications, surgeries, or other interventions). Individuals who are highly dependent on their caregivers, such as young children, those with cognitive disabilities, and the frail elderly, are at particularly high risk for undetected abuse or for being unable to successfully report abuse, but anyone can be successfully abused without their knowledge. Induction of illness via poisoning can be easily unsuspected by highly capable individuals, for example.

When illness falsification starts during pregnancy or young childhood, the victims lack a framework from which to identify abnormal behavior in caregivers. They are subtly or directly rewarded for behaving in ways that are congruent with the story of illness and punished if they intentionally or unintentionally thwart the abuser (Awadallah, et al., 2005; Gregory, 2003). They learn to misperceive normal bodily sensations as dangerous or pathological; they lose trust in their perceptions (Barsky, 1992; Libow, 1995). Children as young as 3 years old have been found to knowingly or unknowingly participate in deceitful reporting of false symptoms (Libow, 1995).

Some victims may suspect abuse but are unwilling to risk disruption of their relationship with their abuser by asking for help (Faedda et al., 2018; Gregory, 2003). Few victims identify abuse and attempt to alert others. Unsuccessful attempts to report MBP abuse can serve to confirm the victim’s fears they will not be believed or decrease their trust in their own perceptions.

MBP abuse can be extremely damaging. Victims may experience physical consequences, including pain, suffering, scars, medical complications, decreased mobility, and death. Developmentally, they may suffer educational neglect and have limited social interaction opportunities. Emotionally, they often incorrectly see themselves as more ill, impaired, and at risk for early death than is factually true. Many suffer from medical trauma due to invasive or painful medical interventions and the belief that they are in danger of dying. Some develop disordered eating due to behavioral conditioning and iatrogenic gastrointestinal dysfunction. They may have or develop suicidal ideation, depression, anxiety, or other psychiatric disorders (Libow, 1995). They are often victimized in other ways as well, including physical abuse, neglect, sexual abuse, and/or emotional abuse. Survivors often have difficulty distinguishing what is real, especially regarding bodily symptoms, and, as adults, may be less likely to seek medical attention (Libow, 1995). Survivors who become aware of their abuse history may no longer trust medical professionals or others who failed to identify abuse and who, with awareness or not, participated in the abusive deception. The development of disrupted, unhealthy attachments is the norm. While further study is needed, case studies (Libow, 2002; McGuire & Feldman, 1989) and a review by Abraham-Bizot et al. (2023) of 29 MBP victims provide preliminary evidence to suggest that up to 50% of childhood MBP abuse survivors falsify illness in themselves later in life.

### Secondary Victims

The friends, family, and professionals who were successfully deceived by the abuser are secondary victims. Upon discovering that they were victims of abusive deception, these secondary victims often feel a traumatic sense of interpersonal betrayal. Especially if they participated in the false narrative put forth by the abuser, they might also experience shame, guilt, anxiety, grief, depression, rumination, sleep disturbance, low energy, gastrointestinal disturbance, or other symptoms that persist over weeks or years (Bursch et al., 2021a, 2021b). They might not know who they can safely speak with due to worries about judgment or legal risk. They can subsequently distrust their ability to accurately perceive others, impacting their ability to develop intimate and trusting relationships.

## STORIES Framework

False stories form the cornerstone of MBP abuse. As described above, these false stories are highly damaging to survivors at a variety of levels. Optimal recovery from MBP abuse requires a variety of interventions, with an emphasis on using accurate information to help the survivor better understand reality and develop a healthier self-narrative.

**Fig. 1:**
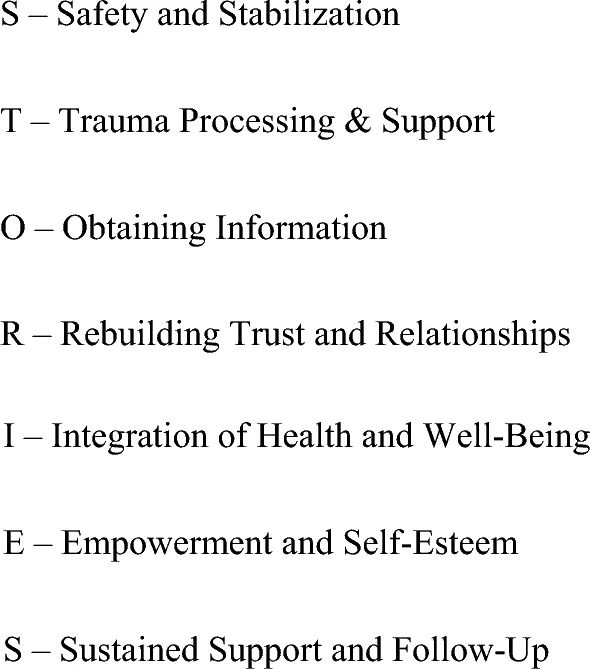
STORIES framework

Following is a heuristic framework to assist professionals who are designing or implementing treatment plans for survivors of MBP abuse, using the acronym STORIES (see Fig. 1). These treatment planning components, which address specific emotional, psychological, and relational needs, are largely intended to be delivered concurrently, not sequentially. However, “safety and stabilization” is the crucial first phase required for the effective provision of trauma-sensitive medical and psychological care. Clinicians are encouraged to use those components of this framework that they find most applicable to the situation before them. While an acronym for this framework is presented to trigger clinicians’ thoughts about helpful interventions and comprehensive care, the components are overlapping.

The treatment components in the STORIES framework apply to both child and adult survivors, and some components apply to secondary survivors as well as primary survivors.

### S—Safety and Stabilization

The highest priority is to provide safety for the MBP survivor and others. Child or dependent adult survivors are often separated from the suspected caregiver, with the survivor being placed in a protected environment (such as a hospital or alternate home environment), to help determine if the symptoms are associated with the suspected caregiver’s presence. If available information suggests or proves MBP abuse, this separation may continue. Ideally, the survivor eventually feels secure and supported in the new environment, with interpersonal predictability and consistency. However, it can take months or longer before a MBP survivor feels safe and supported. In the meantime, survivors can experience confusion, grief, anger, fear, trauma symptoms, depression, suicidal ideation, developmental regression, behavioral disturbance, physical symptoms of stress, or other acute problems.

Survivors of MBP may not be capable of making substantial progress in psychotherapy until they feel safe and secure. Still, they can benefit from slowly building a trusting relationship with a supportive therapist. The frequency and nature of the survivor’s ongoing exposure to the abuser (or possible surrogates of the abuser) can significantly impact the survivor’s sense of safety and treatment progression. Guidance related to the establishment of a safe placement and safe visitation with the abuser is available (Bursch, 2018; Sanders & Ayoub, 2018). Even if well protected, some survivors feel unsafe if they believe that the safety parameters are temporary.

The safety of others, including the perpetrators, should also be considered. For example, while published data on MBP abuser suicidality is scant, perpetrators may be at increased risk for suicide, especially if they also suffer from a Cluster B personality disorder (Abdurrachid & Gama Marques, 2022; McClelland et al., 2023; Vennemann et al., 2006) and should be assessed and provided with appropriate treatment and safety measures. Alternate caregivers of the survivor may require a restraining order due to harassment by the perpetrator. Friends, family, and professionals who were deceived may require safety checks, clinical intervention to address betrayal trauma, or protection due to unwarranted judgments about their level of conscious duplicity or failure to protect (Bursch et al., 2021a, 2021b).

### T—Trauma Processing & Support

Trauma processing includes confronting, understanding, and integrating traumatic experiences to reduce their emotional impact and support healing and recovery. It is an important part of psychotherapy for most MBP survivors, which can include children, adult survivors of childhood abuse, or adult survivors (primary or secondary). Although there are no empirically tested treatment approaches specifically designed for MBP survivors, the larger abuse literature provides evidence-informed options for the therapist to consider based on clinical presentation as well as clinician expertise.

Trauma-Focused Cognitive Behavioral Therapy (TF-CBT), Eye Movement Desensitization and Reprocessing (EMDR), Parent–Child Interaction Therapy (PCIT), Narrative Therapy, and/or Play Therapy are some of the empirically tested treatment approaches which can be implemented to directly process traumatic experiences or support trauma processing depending on the survivor’s age, developmental ability, and circumstance (Gkintoni et al., 2024). Additional treatment interventions are also described below, based on the forensic and clinical experience of seasoned MBP experts who serve on the APSAC’s MBP Committee.

For maltreated children, TF-CBT has the best research support related to effectiveness (Bennett et al., 2021; Gkintoni et al., 2024). In addition to directly processing traumatic experiences, TF-CBT can help verbal children and adult survivors recognize cognitive distortions and reframe negative beliefs.

EMDR, initially used only with adults, has now been adapted for children as young as 6 years old. It has also been adapted for those with intellectual disability (Schipper-Eindhoven et al., 2024). It aims to reduce trauma symptoms by using a dual attentional focus approach that includes guided eye movements (or other bilateral stimulation) while also reprocessing distressing experiences to integrate traumatic memories into a broader neural network and reduce their emotional impact (Torres-Giménez et al., 2024).

While PCIT is not considered a trauma-specific treatment because it does not include the processing of past trauma experiences, it can support children (aged 2–7 years) who have behavioral challenges with their trauma processing by strengthening the caregiver-child relationship, creating a sense of safety, and teaching emotional regulation through consistent, nurturing interactions (Zhang et al., 2024). In such situations, the PCIT therapy is conducted with the survivor and new caregiver (or with the offender if reunification is expected). For children with complex trauma or PTSD, PCIT may be used in combination with or as a precursor to trauma-specific treatments like TF-CBT to make trauma processing more effective.

All survivors with cognitive capacity can benefit from support in developing a coherent narrative of their experience, which can facilitate understanding and emotional release (Raeder et al., 2023; Siehl et al., 2021). Narrative therapy can be used to help survivors re-make their story, separating themselves from the trauma and focusing on one’s strengths, resilience, and abilities to overcome challenges. This can help reduce feelings of shame, guilt, or helplessness, and empower the survivor to take control of their own narrative. For children, narrative therapy can involve using fictional stories, metaphors, art, bibliotherapy, or other creative tools to express and process feelings. Both TF-CBT and EMDR also include narrative creation.

Play therapy can help victims process trauma by allowing them to safely express and work through difficult emotions and experiences through symbolic play (Parker et al., 2021). Like PCIT, it can be used concurrently or as a precursor to trauma-specific therapy. It may be particularly helpful for younger children, or for those with limited verbal or cognitive capacity, to process their experiences via therapist-directed or child-centered and therapist-supported play.

### O—Obtain Information

Because the cornerstone of MBP abuse includes false stories, it is crucial to address these false stories within a therapeutic setting with access to more accurate information. Exposure to more accurate information is especially helpful as survivors develop their healthier self-identity and life narrative. One way to obtain more accurate information is to obtain copies of records or reports that summarize the evaluation that led to the detection of illness or disability falsification. For survivors who have abstract reasoning skills (usually starting around 11 or 12 years of age in typically developing individuals), such records can be highly valuable to integrate into the therapy process. The role of the therapist is to be neutral and curious. The therapist can help by asking questions (such as “What do you remember from this event?” or “Why do you think it has been such a long time since you have had that happen to you?”) and by gently pointing out story discrepancies for the survivor to consider.

Another way to obtain more accurate information is via discussions between the survivor and trusted others, such as the abuser (if the abuser has made meaningful progress in psychotherapy), or another trusted caregiver or professional with access to the facts. Cardona and Asnes (2019) discuss the importance of medical providers acknowledging if they had been drawn into the deception and helping to explain the medical reality that they have come to believe. The authors discuss strategies for doing this, such as having a “staged” or gradual disclosure of information based on the survivor’s ability to understand and readiness to hear the information and with the support of informed caretakers. A trauma-informed approach supports providing the basic facts and then letting the survivor set the pace of further disclosure by asking questions.

An effective apology can be reparative. Effective apologies from an abuser include a detailed account of the abusive behavior, an acknowledgment of responsibility for engaging in the abusive behavior, recognition of the pain and suffering caused by the abuser, communicating that the behavior was not acceptable and that it is now regretted, and a commitment to behaving safely in the future. It is sometimes helpful to explain why the offender was driven to act in this manner if the explanation does not diminish the abuser’s sincere expression of responsibility and remorse. It can also be helpful for the abuser to validate the previously negated perceptions and experiences of the survivor, and to provide the true facts in place of lies that were told. Apologies from those who unknowingly participated in the false narrative, such as friends, family members, or professionals, can follow a similar format related to behavior they now regret. Importantly, the survivor should not be asked to forgive the perpetrator or others.

As therapy progresses, some survivors benefit from reaching out to a wider range of witnesses of their life to learn about their observations and thoughts. This can include family friends, more distant relatives, previous school personnel, child protective services workers, or past health providers, as examples.

Please see the Supplementary File for additional details designed to assist psychotherapists, especially those working with survivors having difficulty acknowledging past abuse.

### R—Rebuilding Trusting Relationships

Along with a safe and secure living environment, psychotherapy can be helpful in the process of rebuilding trust and relationships. In cases where the survivor is not sure what to believe, a neutral and curious stance is essential to maintain so that the psychotherapist is not perceived to be taking a stance against the abuser. That does not mean agreeing with statements made by the survivor that are untrue, but it does mean refraining from trying to convince the survivor of their abuse. The goal is to support the perceptions and discovery of the truth by the survivor over time.

Psychotherapists and caregivers can assist survivors rebuild trusting relationships if they can provide stability, dependability, a nonjudgemental stance, empathy for both the abuser and the survivor(s), good boundaries, patience, warmth, collaboration, confidentiality, and active listening. Therapists and caregivers alike can expect to be tested by survivors. For example, they might test the relationship by pushing boundaries, disclosing sensitive information to assess reactions, or lying. It can be helpful to ensure that caregivers are aware of this normal part of rebuilding trusting relationships so that they are prepared to stay the course during these challenges.

If progress has not been made by the abuser in psychotherapy, limited safe access to the abuser in a therapy session can help some teens or adults observe the ongoing destructive behaviors of the abuser from a new perspective. This can serve to help the survivor alter their life narrative with the new knowledge of the deficits of the abuser. However, these encounters should be terminated once the value of these observations is diminished or if it is traumatizing to be exposed to the abuser.

Due to the risks associated with family placements, many child survivors are placed in foster care homes with previously unknown individuals while efforts are made to rehabilitate the abuser. Some child survivors are permanently removed from the care of their abusers. Foster parents can help build a trusting relationship with the child by involving the child in family activities, encouraging them to decorate their space, and obtaining their input when making decisions about things like meals or family outings. These efforts can help the child feel valued and that they are accepted by the family. Because MBP differs from other forms of abuse, foster parents benefit from education on this topic, protection from the abuser, a detailed description of the abuse experienced by the child, information about genuine medical problems that are being addressed versus falsified problems, and a parenting plan that helps them make good decisions in the moment, such as when they observe sick role behavior, or if they have an unexpected encounter with the abuser. Some survivors “adopt” their foster families for the rest of their lives, remaining in close contact with them even after emancipation from the child welfare system.

### I—Integration of Health and Well-Being

Survivors benefit from physical, developmental, and psychological care, via a multidisciplinary team approach. Team composition varies depending on the needs of the survivor and available resources. Individuals who might be on a team include a primary care physician, needed medical specialists, psychotherapists, physical or occupational therapists, feeding specialists, primary caregivers, and/or child or adult welfare workers. This team typically starts with developing a rehabilitation plan designed to optimize health and functioning.

Unnecessary medical treatments should be discontinued or weaned off under close medical supervision. Whenever possible, a sequential approach is recommended so that any regressions in symptoms are easier to understand and address appropriately. For example, ideally, only one medication should be discontinued at a time so that changes in medical status can be attributable to that treatment change. Some medical interventions might require a very slow weaning process if there are iatrogenic consequences to the intervention that require management or if the survivor is required to cooperate with the change but is not yet willing. An example is slowed gut motility due to malnutrition in children who believe they are dependent on a g-tube and unable to eat by mouth. Interventions targeting both the slowed motility and the child’s confidence in eating by mouth may be required to successfully remove a g-tube. Likewise, a child who incorrectly believes she has lethal allergies may naturally have great fears about trying previously forbidden foods.

Physical or occupational therapy can aid with improved physical functioning. In some cases, increased social opportunities and appropriate academic settings can result in survivors gaining years of developmental milestones within months. Children or young adults often respond to a positive reward plan for improved functioning. Exposure therapy techniques may be needed for those needing to overcome their fears, such as fears (based on falsification) of having a severe allergic reaction, giving up a g-tube or wheelchair, or going to school. Sick behavior should be briefly evaluated by the caregiver but then ignored if benign. Caregivers help survivors by focusing discussions and attention on health and functioning, and by encouraging independence and individuation. Excellent clinical guidance for therapists treating youth with chronic pain, functional neurological disorders, or other ongoing functional symptom disorders is available (Williams & Zahka, 2017). Older survivors may also require life skills training, such as grocery shopping, meal planning, cooking, laundry, budgeting, resume writing, job interviewing, and driver’s education.

### E—Empowerment and Self-Esteem

Being consistently told by an abuser you are wrong or confused, and that you cannot trust your perceptions or experiences, can have devastating effects on a survivor’s mental health. Along with questioning one’s sense of reality and beliefs, survivors may feel isolated and powerless. Survivors often have feelings of inadequacy, disorientation, self-doubt, guilt, or shame. They may feel unworthy of love and struggle with self-esteem and self-worth. Therapy goals can include cultivating self-compassion, helping them realize they are not responsible for the abuse, and encouraging them to trust themselves and others. The therapist can also help the survivor identify negative self-talk based on cognitive biases and teach the survivor to replace those thoughts with more helpful thoughts.

An important goal of the rehabilitation plan is to provide opportunities for the survivor to develop confidence and a positive self-image. Engaging in goal-setting and decision-making can be a new experience for some previously highly controlled survivors. For example, some survivors have never had the opportunity to choose what to wear or what to eat for lunch.

### S—Sustained Support and Follow-Up

Survivors can expect to revisit their past at various times in their lives, especially during important life transitions or milestones. As mentioned earlier, having one’s own child can bring up feelings of anger or incredulity. Seeing childhood friends or previously estranged family members might trigger new memories or trauma symptoms. It can be helpful for this trajectory to be normalized.

For older survivors, therapists should encourage participation in survivor support groups or individual peer networks. Connecting with others who have gone through similar experiences can help validate the survivor’s feelings, reduce isolation, and foster a sense of community.

## Limitations

Please note that the heuristic STORIES framework has not been scientifically validated. While the framework includes best practices recommendations for maltreated individuals and evidence-based treatments for trauma and behavioral challenges, it also includes recommendations based on the clinical experiences of the authors and their expert colleagues. Due to significant barriers to systematically studying MBP abusers and survivors, clinical and forensic experience and published case studies form the cornerstone of the MBP specific information presented.

## Data Availability

No datasets were generated or analysed during the current study.
